# COVID-19 in pastoral contexts in the greater Horn of Africa: Implications and recommendations

**DOI:** 10.1186/s13570-020-00178-x

**Published:** 2020-10-13

**Authors:** Evan F. Griffith, Loupa Pius, Pablo Manzano, Christine C. Jost

**Affiliations:** 1 Cummings School of Veterinary Medicine, Tufts University, North Grafton, USA; 2 Dynamic Agro-Pastoralist Development Organization (DADO), Kaabong, Uganda; 3 Arid Landscape Initiative (ALIN Africa), Moroto, Uganda; 4 Coalition for European Lobbies on Eastern Africa Pastoralism (CELEP), Brussels, Belgium; 5 Global Change and Conservation Lab, Organismal and Evolutionary Biology Research Programme, Faculty of Biological and Environmental Sciences, University of Helsinki, Helsinki, Finland; 6 Helsinki Institute of Sustainability Science (HELSUS), Faculty of Biological and Environmental Sciences, University of Helsinki, Helsinki, Finland; 7 Global Health Support Initiative III, Social Solutions International, United States Agency for International Development Bureau for Humanitarian Assistance, Washington, D.C., USA

**Keywords:** COVID-19, Public health, Greater horn of Africa, Community one health teams, Food security, Livelihood security, Gender

## Abstract

COVID-19 is a global pandemic that continues to spread around the world, including to Africa where cases are steadily increasing. The African Centres for Disease Control and Prevention is leading the pandemic response in Africa, with direction from the World Health Organization guidelines for critical preparedness, readiness, and response actions. These are written for national governments, lacking nuance for population and local differences. In the greater Horn of Africa, conditions unique to pastoralists such as inherent mobility and limited health and service infrastructure will influence the dynamics of COVID-19. In this paper, we present a One Health approach to the pandemic, consisting of interdisciplinary and intersectoral collaboration focused on the determinants of health and health outcomes amongst pastoralists. Our contextualized public health strategy includes community One Health teams and suggestions for where to implement targeted public health measures. We also analyse the interaction of COVID-19 impacts, including those caused directly by the disease and those that result from control efforts, with ongoing shocks and vulnerabilities in the region (e.g. desert locusts, livestock disease outbreaks, floods, conflict, and development displacement). We give recommendations on how to prepare for and respond to the COVID-19 pandemic and its secondary impacts on pastoral areas. Given that the full impact of COVID-19 on pastoral areas is unknown currently, our health recommendations focus on disease prevention and understanding disease epidemiology. We emphasize targeting pastoral toponymies with public health measures to secure market access and mobility while combating the direct health impacts of COVID-19. A contextualized approach for the COVID-19 public health response in pastoral areas in the Greater Horn of Africa, including how the pandemic will interact with existing shocks and vulnerabilities, is required for an effective response, while protecting pastoral livelihoods and food, income, and nutrition security.

## Introduction

Coronavirus disease (COVID-19) is an infectious disease of humans caused by a coronavirus newly discovered in 2019—SARS-CoV-2. COVID-19 has been declared a global pandemic by the World Health Organization (WHO) and continues to spread around the world. Amongst the Intergovernmental Authority of Development (IGAD) countries in the Greater Horn of Africa (GHA), 46,620 cases have been reported with 1293 fatalities as of 21 July 2020 (IGAD 2020). The response to COVID-19 in Africa is being led by the Africa Centres for Disease Control and Prevention, a specialized technical institution of the African Union. It is directed in part by WHO guidelines for critical preparedness, readiness, and response actions (PRRAs) for COVID-19 (World Health Organization 2020). However, these guidelines are intended for national governments, and not specific to any population.

**Fig. 1:**
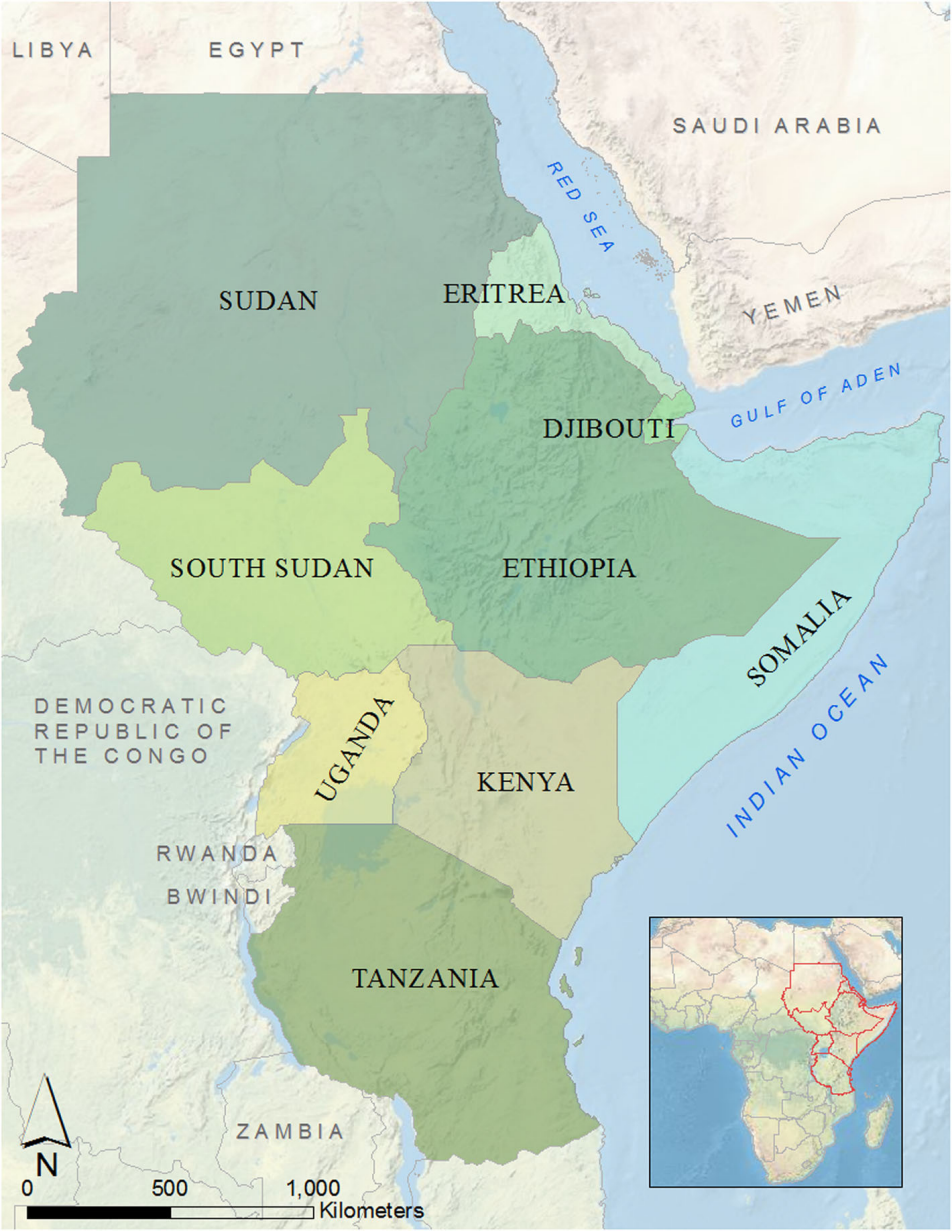
The Greater Horn of Africa region. Created by EFG with ArcMap 10.6.1. Data sources: ArcGIS, US National Park Service

Human health programmes often fail to reach pastoralists in the GHA due to their inherent mobility and marginalization, and the limited health infrastructure in the region’s arid and semi-arid lands (ASALs) (Dubale and Mariam 2007; Griffith et al. 2020). Mobility is not only a defining and differential feature of pastoralist livelihoods, but it is also essential to the present and future viability of livestock production in most of the world’s drylands (de Jode 2010; Nugteren and Le Côme 2016; Manzano and Salguero 2018; Molina-Flores et al. 2020). Considering that mobility restrictions have proven to be the most effective measure to control the spread of COVID-19, the management of the pandemic amongst pastoralists is likely to be particularly challenging (Kissler et al. 2020). Moreover, the impacts of COVID-19 interventions (e.g. economic disruption due to controlled shutdowns of non-essential business activities and constraints on supply chains for goods and services) will interact with and compound ongoing shocks in the region, threatening pastoral livelihoods and food, income, and nutrition security. It is therefore necessary to develop contextualized public health strategies and examine how pastoral livelihoods can be protected in the face of the COVID-19 pandemic. In this paper, we define the GHA as comprising nine countries: Ethiopia, Somalia, Eritrea, Djibouti, Kenya, Uganda, Sudan, South Sudan, and Tanzania (Fig. 1).

Pastoralism is a livelihood and a food production system found all over the world’s rangelands and seasonally on large areas of farmlands. Primarily a herding system, pastoralism has a unique ability to convert non-human-edible proteins unpredictably distributed throughout highly variable ecosystems into meat, milk, livelihoods, and income (Food and Agriculture Organization of the United Nations 2011). For this reason, it can be described as a specialization to take advantage of environmental variability (Ministry of State for Development of Northern Kenya and other Arid Lands 2012). While the GHA has a young age structure with relatively few people older than 65 years old, much of the traditional ecological knowledge that livestock productivity relies on in these systems is held by community elders (Oba 2012; United Nations Economic Commission for Africa 2015)—a particularly relevant fact for a disease-like COVID-19 affecting mostly old people (Lloyd-Sherlock et al. 2020). According to the United Nations Food and Agriculture Organization (FAO), over 268 million people rely on pastoralism as a livelihood (Food and Agriculture Organization of the United Nations 2018). But the number of those who benefit from pastoralism is much larger, as these systems supply important value chains, provide critical ecosystem services, and contribute to maintaining landscape functionality (Hoffmann et al. 2014). Pastoralism is particularly widespread in the GHA because of the economic advantage that milk-based economies historically provided in ASALs and the integration of camels in some areas as a species capable of longer lactation during dry seasons (Gebremichael and Girmay 2019; Marshall 1990; Nori et al. 2006). The importance of milk is sustained for the long-term upcoming trends in the region (Nori 2010; Manzano and Yamat 2018). In the GHA ASALs, pastoralism is practised extensively and makes contributions to national gross domestic products (GDP)—19%, 13%, 8%, and 7.5% in Ethiopia, Kenya, Uganda, and Tanzania, respectively, and 80% of agricultural GDP in Sudan (African Union 2013; Nyariki and Amwata 2019). It is also critical for food security, providing milk to remote populations and 90% of the meat consumed in East Africa (Nyariki and Amwata 2019). Pastoralists in Sudan and Somalia are major exporters of livestock to the Gulf States (African Union 2013).

COVID-19 transmissibility is directly related to population density and mobility, as both of these factors increase contacts between infectious and susceptible people (Fang et al. 2020; Rocklöv and Sjödin 2020). The low population densities common to pastoral areas could reduce COVID-19 transmissibility, but movement inherent to pastoralism increases mixing and contact, thereby increasing transmissibility. Markets and population centres likely pose the highest risk to pastoralists and can provide a source of infections that can introduce COVID-19 to pastoral lands. Thus, the COVID-19 response should focus on preventing transmission in these locations, while also protecting pastoral livelihoods from the secondary effects of disease control efforts.

It is important to centre COVID-19 prevention and control efforts on understanding its epidemiology in pastoral contexts. Diagnostic testing should also be emphasized in the early stages of the outbreak to guide prevention and control strategies (Nkengasong and Mankoula 2020). Lessons from the Ebola outbreak in West Africa can contribute to tracking the spread of COVID-19 in pastoral areas, specifically through phylodynamic analysis of how the epidemiology and immunology of a virus interact to shape its evolution to help estimate the effective reproductive number of the disease (du Plessis and Stadler 2015; Lai et al. 2020; Stadler et al. 2014).

In this paper, developed in part through an expert-opinion elicitation, we discuss the components of a One Health approach to the COVID-19 pandemic, with a focus on disease epidemiology, prevention, and protecting food, income, and nutrition security and pastoral livelihoods (Table 1). One Health aims to promote human, animal, and environmental health through multidisciplinary and multisectoral approaches. Zoonotic disease has predominantly been at the centre of One Health, leading to the collaboration between human and animal health sectors. Yet, this narrow application fails to fully address the social, environmental, and economic drivers (i.e. determinants) of health. Fully realizing the potential of One Health requires expanding partnerships across economic, environmental, and health sectors, an approach that is increasingly being adopted in the GHA (Vétérinaires Sans Frontières International 2020). Thus, for the purposes of this paper, a One Health approach to the COVID-19 pandemic can be thought of as an interdisciplinary and intersectoral collaboration focused on the determinants of health and health outcomes amongst pastoralists that take into account the ongoing shocks and vulnerabilities in the GHA. The goals of this strategy include limiting the spread of COVID-19 in pastoral areas, especially amongst elders, while protecting food, income, and nutrition security and pastoral livelihoods. We do not describe a one-size-fits-all solution to COVID-19 or address the entirety of the medical or public health response in GHA pastoral contexts but rather describe locally appropriate general principles of COVID-19 prevention and epidemiology based on previous research and experience.

**Table 1:** Experts consulted for this paper and their affiliations

Name	Affiliation
Sadia Mussee Ahmed	Pastoralist and Environmental Network in the Horn of Africa (PENHA), Somalia
Abdirahman Mohamed Ali	Deutsche Gesellschaft für Internationale Zusammenarbeit (GIZ) GmbH Somalia
Esmael Tessema Ali	Vétérinaires Sans Frontières (VSF) Germany
Hellen Amuguni	Cummings School of Veterinary Medicine, Tufts University, USA
Rupsha Banerjee	International Livestock Research Institute (ILRI)
Jeanne Coffin-Schmitt	Cornell University, USA
Anthony Egeru	Makerere University/Food for Africa Development Agency (Afrifood)
Emmanuel Emaruk	VSF Belgium
Fiona Flintan	ILRI/International Fund for Agricultural Development (IFAD)/International Land Coalition (ILC)
Echi Christina Gabbert	Goettingen University, Germany/Lands of the Future Initiative
Margherita Gomarasca	VSF International
Anthony Maina Kibata	Welthungerhilfe (WHH)
Tabitha Kimani	United Nations Food and Agricultural Organization (FAO), Nairobi, Kenya
Job Ronoh Kipkemoi	Turkana County Government Directorate of Veterinary Services, Lodwar, Kenya
Saverio Krätli	German Institute for Tropical and Subtropical Agriculture, and Transdisciplinary and Social-ecological Landuse Research (DITSL)
Edward Ole Lekaita	Ujamaa Community Resource Team (UCRT) Tanzania
Jeffrey C. Mariner	Cummings School of Veterinary Medicine, Tufts University, USA
Timothy Njagi	Tegemeo Institute of Agricultural Policy and Development/Egerton University, Kenya
Nyang’ori Ohenjo	Center for Minority Rights Development (CEMIRIDE)
Diana Onyango	VSF Suisse
Peter Ken Otieno	Coalition for European Lobbies on Eastern African Pastoralism (CELEP)/Resource Conflict Institute (RECONCILE)
Merciline Lina Oyier	CELEP/Catholic Organization for Relief and Development Aid (Cordaid)
Tezera Getahun Tiruneh	Pastoralist Forum Ethiopia
Hussein Wario	Center for Research and Development in Drylands, Kenya
Ann Waters-Bayer	CELEP/Agrecol Association for AgriCulture & Ecology
Barbara Weiland	ILRI

## Pastoral public health during COVID-19: Epidemiology and prevention

Public service delivery amongst pastoralists in the GHA is limited by programmes ill-suited to remote, mobile populations and logistical, organizational, and financial constraints (Abakar et al. 2016; Schelling et al. 2015; Zinsstag et al. 2016). Previous examples show that community health workers (CHWs) and community-based animal health workers (CAHWs) are cost-effective and locally available service providers in remote, pastoral areas that can help to overcome these constraints. Community health workers have provided preventative, promotive, and limited curative primary healthcare services for pastoralists and therefore may be able to help with COVID-19 response activities (El Shiekh and van der Kwaak 2015; World Health Organization 2017). Similarly, CAHWs play a substantial role in providing localized animal health services in pastoral areas. Evaluation of CAHW programmes in the GHA found they were trusted, accessible, improved animal health and livelihoods, and played a critical role in emergency response (Leyland et al. 2014). Community-based animal health workers have worked with CHWs previously to provide public health services (e.g. case reporting and promotional activities), including responding to cholera outbreaks in cattle camps in South Sudan (Vétérinaires Sans Frontières Germany 2018). It therefore may be possible for CHWs and CAHWs working together in community One Health teams(COHTs) to contribute to COVID-19 prevention measures summarized in Table 4. These health workers are members of the community, forming a direct link between health professionals and pastoralists, which would allow for the implementation of prevention strategies as pastoralists engage in daily mobility and migration across territorial boundaries. However, CHWs and CAHWs interact with health care structures and providers for reporting and supervisory purposes and in the case of CAHWs resupply drugs and other equipment in market centres. These are settings where the risks of COVID-19 transmission are high, and therefore, COVID-19 surveillance and testing in CHWs and CAHWs should be prioritized.

Community health volunteers (CHVs) and livestock community disease reporters (CDRs) in Turkana County, Kenya, are already being trained on COVID-19 case definitions and WHO and government guidelines towards its prevention to contribute to case reporting and health promotion activities under the guidance of the Turkana County One Health Committee (Kipkemoi Table 1). Important considerations of COHTs with suggested solutions are summarized in Table 2.

**Table 2:** Important considerations of COHTs and COVID-19 public health activities with suggested solutions

Consideration	Solution
Insufficient number of trained and supervised CHWs in remote pastoral areas.	Nomination of community members to be trained by health authorities to report into COVID-19 surveillance systems
Level of training may not consider a highly transmissible disease with heightened mortality rates.	Provide specialized COVID-19 training, including case definitions and public health measures (e.g. physical distancing, personal protective equipment uses) to prevent transmission
Personal health risks COHTs may face given their level of training.	
Circulation of COHTs may contribute to the spread of COVID-19.	
The decision to involve CHWs and CAHWs/CDRs in the COVID-19 response is a combined national/local policy decision.	Form One Health coordination structures (e.g. One Health Task Force, One Health county/subcounty committee) based on existing government organization with national/local authority to implement COVID-19 activities

Similar to the One Health Committee in Turkana, a Regional One Health Task Force in the Somali and Oromiya regions of Ethiopia is taking the lead in COVID-19 preparedness and response activities (Onyango in Table 1). These examples, in addition to what is presented in Table 3, illustrate One Health coordination structures that can contribute to the pandemic response through addressing livelihood, food, income, and nutrition security and health concerns.

**Table 3:** Examples of a One Health approach in pastoral areas in the GHA (Griffith et al. 2020; Kamadjeu et al. 2015; Lankester et al. 2019; Mtui-Malamsha et al. 2020; Musoke et al. 2016; Ndejjo et al. 2019; Onyango et al. 2019; United Nations Environment Programme and International Livestock Research Institute 2020)

Institutions/developmentpartners	Team composition	Location	Activities
Ministry of Health, Ministry of Livestock, United Nations Children’s Fund, WHO, and FAO	Human and animal health, community elders	Somalia	Joint human and animal vaccination
Ministry of Livestock, Agriculture, and Fisheries and Ministry of Health	Zoonotic Disease Unit, County One Health Units	Kenya	Active collaboration at the animal, human, and ecosystem interfaces towards better prevention and control of zoonotic diseases
Afya Timiza, Amref Health Africa-Kenya, and Turkana County Government (Ministry of Health and Sanitation; Ministry of Agriculture, Pastoral Economy, and Fisheries)	Kimormor programme: human and animal health, public and emergency services	Kenya	Health services (e.g. child vaccination), National Health Insurance Fund/livestock-based insurance, birth registration, financial services, food distribution, veterinary/agriculture
District Medical Office and District Veterinary Office	Human and animal health	Tanzania	Soil-transmitted helminths and canine rabies prevention and control activities
One Health Coordination Desk (Office of the Prime Minister); Ministry of Livestock and Fisheries; Ministry of Health, Community Development, Gender, Elderly and Children; FAO; and Sokoine University of Agriculture	One Health rapid response teams: human and animal health, environment, wildlife, disaster management	Tanzania	Disease outbreak investigations, field investigations and interventions (e.g. rabies and anthrax in humans and animals)
Vétérinaires Sans Frontières - Suisse, International Livestock Research Institute, Medical Collaboration Committee, national governments	HEAL Project: One Health for Humans Environment Animals and Livelihoods	Southern Ethiopia, northern Kenya, and Somalia	Establishment of One Health Units for the integration of human health, animal health, and rangeland management service provision
Ministry of Health – Uganda; Commissioner Environmental Health, regulated by Allied Health Professionals Council	Environmental health practitioners	Uganda	Inspecting livestock before slaughter as well as the meat in slaughterhouses and butcheries; monitoring the destruction of condemned meat; investigating zoonotic disease outbreaks and monitoring disease control programmes; ensuring the control of disease vectors and vermin; providing communities with health education on pertinent issues; food safety; helping to enforce Uganda’s public health legislation

The WHO prerparedness, readiness and response actions include risk communication and community engagement, surveillance, case finding, contact tracing and management, and public health measures (World Health Organization 2020). Community engagement has been conducted in pastoral areas through participatory community dialogue with key influencers (Table 4). Participatory community dialogue has been used in a variety of processes including rapid needs assessments, disease outbreak investigations, and awareness and sensitization (Vétérinaires Sans Frontières Suisse 2019). It can also help to build trust and involve communities in the development of socially acceptable and beneficial interventions and development (Grillos 2014). Communication infrastructure is limited in pastoral areas of the GHA, posing a challenge to risk communication. We provide examples of communication tools and methods that can be utilized for COVID-19 risk communication in Table 4.

**Table 4:** Contextualized WHO critical preparedness, readiness, and response actions in pastoral areas in the GHA

WHO PRRAs	Approach
Community engagement	Participatory community dialogue with community members, leaders and platforms: • Uganda: kraal leaders, opinion leaders, local council members, council of elders (Akiriket), traditional healers, village health teams, and CAHWs • Kenya: community elders, women groups, youth groups, chiefs, Member of County Assembly, ward administrators, traditional healers, CHWs, and community disease reporters • Ethiopia: community leaders, religious leaders, multi-stakeholder innovation platforms, women, generation-and age-set platforms (such as Gadaa), youth groups, traditional healers, CHWs, and CAHWs • South Sudan: non-governmental organizations (NGOs)/community-based organizations (CBOs), youth groups, village chiefs, headmen, traditional healers, CHWs, and CAHWs • Somalia: community associations, local NGO consortiums, religious leaders, village development committees, traditional healers, CHWs, and CAWHs • Tanzania: traditional leaders, village leaders, chiefs, woman rights and leadership forums, youth groups, traditional healers, CHWs, and CAHWs • Sudan: Arab tribes—Nazirs, Ummdas, and Sheikhs; agro-pastoralists (Fur) in Darfur—Farsha, Damagnawi, and Shurtai; pastoralist union/association; livestock corridor management committees; local peace councils; and spiritual and traditional healers Communication methods: • Social media (e.g. WhatsApp, Facebook, Telegram, Instagram) • Local telecommunication companies (e.g. MTN, Airtel, Vodocom, Saraficom, Ethio Telecom, Halotel, TTCL, Somtel, Telesom)—customize different voice messages in different languages, bulk messaging • Local and community radio stations (e.g. talk shows and spot messages)—identify speakers/local groups/individuals to deliver the right message • Traditional information nodes • Traditional dramas, poems, songs, sermons
Surveillance, case finding and contact tracing	• Investment in COHT training and resources • Mobile phone-based syndromic surveillance • Participatory surveillance to identify cases and understand the epidemiologic situation • Traditional healers (case finding)
Case management	• PCD to develop community-appropriate isolation and quarantine measures building on previous human and zoonotic disease outbreaks (e.g. cholera)
Public health measures (PHMs)—focus on high transmission risk areas and behaviours	• Behaviours: ◦ Greeting and farewell protocols ◦ 1 m between people ◦ Discourage communal food, utensil, and tobacco sharing • Physical marketplaces (USAID Bureau for Resilience and Food Security 2020): ◦ Expand physical market space ◦ Entrance and exit protocols ◦ One-way flow if possible ◦ Body temperature checks ◦ Clean and disinfect physical space ◦ Ensure handwashing facilities (e.g. upgrade water supply or use tippy-taps) ◦ Cloth face coverings (respiratory etiquette) ◦ Prominent public health messaging on how to prevent transmission ◦ Leverage digital tools to facilitate payment and delivery ◦ Encourage only one family member to go to the market • Livestock physical marketplaces (USAID Bureau for Resilience and Food Security 2020): ◦ Expand the area of sale yard and space out vendors ◦ Encourage small sales areas adjacent to the main market yard ◦ Limit the number of market participants to those buying and selling animals ◦ Mechanisms to bypass physical markets (e.g. online or through cell phones) ◦ Ensure handwashing facilities (e.g. upgrade water supply or tippy-taps)

Community health workers on One Health teams, and where appropriate CAHWs, can be trained to report into COVID-19 surveillance systems, particularly mechanisms such as hotlines that One Health Task Forces have established for emergency COVID-19 reporting. In five northern Kenyan counties, CDRs (and other animal health agents) play an active role in surveillance systems through the use of smartphone apps linked to a cloud server that generates disease trends that can be used to inform targeting of response interventions in space and time (Bodha et al. 2017; CGIAR 2018; Long’or et al. 2018). In Tanzania, the One Health *AfyaData* app was trialled in Morogoro and Ngorongoro districts by CHWs and professionals in the public and animal health fields for disease reporting and included a One Health Knowledge Repository that provided users with information on human and animal diseases such as case definitions (Karimuribo et al. 2017). Traditional healers can also contribute to case finding, if given the appropriate training and support (Sima et al. 2019).

Participatory epidemiology practised by animal and public health professionals has been used in multiple settings to elucidate the epidemiology of emerging and transboundary diseases (Bett et al. 2015; Jost et al. 2010; Mariner et al. 2012, 2014; The International Livestock Research Institute 2011; Walker et al. 2015). A similar approach, in concert with phylodynamic analysis and diagnostic testing described previously, can be implemented by One Health coordination structures responding to the pandemic to identify potential outbreaks of COVID-19 and understand disease epidemiology as it occurs in pastoral settings. However, the risks of surveillance and data collection methods such as these, which depend on outside experts visiting pastoral communities and inadvertently introducing COVID-19 while asymptomatic, should be kept in mind; public health measures put in place to limit these risks; and preference given whenever possible to approaches such as community One Health teams and remote data collection that do not bring outsiders into contact with at-risk communities.

Pastoral toponymies, including markets and water points, are high transmission risk areas. It is therefore necessary to implement public health measures in relation to these areas, including hand hygiene to reduce the risk of surface/fomite transmission, and cloth face covering distribution to reduce the risk of aerosol/airborne transmission at gathering points (Table 4). The availability of water and soap at markets makes this a feasible strategy, while investments in public health measures at other toponymies should be emphasized by the governments and response partners. Equally important are high transmission risk behaviours like greetings and communal food sharing. Public health measures, including hand hygiene, respiratory etiquette, and social distancing, can also help to decrease the risk of these behaviours (Table 4). Community One Health teams can implement public health measures through health promotion activities. In addition, participatory community dialogue can be used to develop and implement local context-specific public health measures.

## Pastoral food and livelihood security: Offsetting the secondary impacts of the public health response

Food insecurity and malnutrition are common amongst pastoralists in the GHA, with many contributing factors including: seasonal environmental variation; weak services and infrastructure and shocks e.g. desert locusts, drought, livestock diseases outbreaks, floods, conflict and livestock raiding, food price increases, and market closures. There are also long-term trends (e.g. governance problems, development displacement, and environmental degradation particularly of pastures related to regional climatic shifts) (Catley et al. 2009). These risk and vulnerability factors will interact and compound issues raised by COVID-19 (Table 5).

**Table 5:** Recommendations for how to combat the combined impact of COVID-19 and ongoing shocks and stressors

COVID-19 impacts	Compounding/interacting factor	Implications	Recommendations
Direct mortality and morbidity	• Comorbidities (e.g. tuberculosis) • Conflict and raiding	• Increased morbidity and disease severity • Increased risk of transmission	• COHTs • Contextualized PRRAs (Table 4) • Community-based peace efforts, in concert with resilience building and COVID-19 response
Reduced access to pastoral areas	Desert locusts	• Decreased ability to implement locust control measures • Personal protective equipment limitations • Loss of pasture and crops • Decreased ability to deliver emergency services	• Designate locust control, surveillance, and response activities as essential services, and equipment and supplies as essential goods • Coordinate local, national, and regional COVID-19 and locust control activities • Implement locust surveillance and control activities while adhering to PHMs
	Human/livestock/zoonotic disease	• Less ability to prevent and control disease • Negative impact on human health • Negative impact on animal health • Reduced income due to lower market prices for sick animals, quarantines and loss of public trust	• Continued animal health measures implemented through One Health coordination structures while adhering to COVID-19 PHMs • CAHWs on COHTs provide animal health support in addition to COVID-19 activities while adhering to PHMs • Voucher-based programmes for animal health services
	Floods, drought	• Cannot deliver emergency support	• Designate disaster relief as essential • Implement activities while adhering to PHMs
Reduced mobility (daily grazing, migration)	• Limited land access (e.g. development displacement) • Drought/flooding • Desert locusts	• Increase pressure on rangelands • Negatively impacts livestock health and productivity • Decreased access to resource contributes to conflict	• Identify local mobility norms through community engagement and research (e.g. participatory rangeland management) • COHTs emphasize PHMs at pastoral toponymies • Direct support, such as livestock fodder as needed • Destocking
Reduced access to markets	• Limited market access (e.g. distance to markets) • Livestock disease (market closures)	• Cannot sell livestock products or buy food (e.g. grain) and other essential items	• Keep markets open while implementing PHMs (Table 4) • Harvest natural foods • Food aid • Alternative livelihood development • Traditional methods of preserving livestock products • Selective destocking • Mobile abattoirs • Remote livestock sales using mobile phones

The epidemiology of COVID-19 is influenced by co-morbidities and health status. Pastoralists generally have poor health compared to national averages, with pre-existing acute and chronic health conditions, and widespread malnutrition that put them at increased risk from COVID-19. For example, due to limited access to care, pastoralists in Ethiopia receive inadequate treatment for tuberculosis (Nooh et al. 2019). Tuberculosis infection increases susceptibility to COVID-19 and disease severity (Liu et al. 2020). Further, pastoralist children and women are more at risk of acute malnutrition compared to those in sedentary populations (Schelling et al. 2016). The immune response is weakened by inadequate nutrition, leading to negative impacts of malnutrition in relation to COVID-19 (Caccialanza et al. 2020).

Decreased access to pastoral areas due to COVID-19 control measures (e.g. movement restrictions) will impact desert locust control, human and livestock disease prevention and control, and disaster relief. An ongoing wave of locusts in the GHA has led to the destruction of crops and pasture, with climate conditions expected to result in a dramatic increase in locust numbers during the coming months (Regional Desert Locust Alliance 2020). COVID-19 may limit entry into pastoral areas by locust control personnel and result in personal protective equipment (PPE) “competition,” as both locust control and health activities require PPE. Designation of locust control as essential can ensure access to pastoral areas, while a coordinated response by governments and development partners can help to ensure that PPE specialized for use in pesticide application is available (Regional Desert Locust Alliance 2020). Livestock disease outbreaks negatively impact pastoral livelihoods through morbidity and mortality, decreased production, and market bans (Catley et al. 2009). Thus, control measures for ongoing livestock disease outbreaks in the region (e.g. foot and mouth disease, Rift Valley fever), the peste des petits ruminants eradication campaign, and for endemic diseases of importance like contagious caprine pleuropneumonia are critical (Njeumi et al. 2020; ProMED-mail 2020a, b). Decreased or limited access to pastoral areas by animal health personnel can harm these efforts. Animal health officials can contribute to these efforts through disease surveillance and control measures, while adhering to COVID-19 public health measures. The GHA is also currently experiencing extreme flooding events (FloodList 2020). Emergency support personnel coming from population centres to pastoral areas could act as a source of infection, or movement restrictions could restrict emergency support. Emergency services should be designated essential, and personnel should undertake COVID-19 public health measures during the response.

Pastoralist movement restrictions due to COVID-19 will interact with development displacement and ongoing regional shocks to decrease access to pasture and water and increase pressure on rangelands. These will negatively impact livestock health and productivity. Livestock mobility is driven by livestock nutritional needs and can be thought of as short range (i.e. daily grazing mobility) or long range (i.e. migration), in response to highly variable resource availability and predictable spatio-temporal variation in rangeland conditions, respectively (Turner and Schlecht 2019). Daily grazing and migration should be protected, as these are essential aspects of pastoral livelihoods. Local mobility needs and dynamics can be supported through participatory community dialogue using community planning techniques such as participatory rangeland management or community-based natural resource management committees (Flintan et al. 2018). To support mobility, COHTs can undertake COVID-19 risk communication and community engagement for herders travelling with their livestock. Public health measures at pastoral toponymies (e.g. encampment and water points) should be emphasized. Direct support, such as voucher systems to purchase livestock fodder, can also be implemented when necessary to ensure livestock nutritional needs that may arise because of mobility restrictions. Reduced mobility and resource limitations can contribute to conflict (Avis 2018). This will have additive effects on food security and livelihoods, as conflicts in pastoral areas resulting in asset losses, especially livestock assets, trigger heightened needs for humanitarian assistance and worsen household food insecurity (Catley et al. 2016).

Livestock market closures negatively impact food security and livelihoods (Catley et al. 2009). Pastoralists sell animals when they need cash to purchase food or for secondary expenditures (e.g. health services) (Little et al. 2014). With the closure of livestock markets, already occurring in the GHA due to COVID-19, pastoralists’ ability to buy food will decrease, contributing to malnutrition. This is especially true for poorer households who have greater food purchasing needs (Little et al. 2014). Market access can be preserved through a nuanced approach to COVID-19 that keeps markets open while implementing public health measures (Table 4) (USAID Bureau for Resilience and Food Security 2020). Livestock insurance activation and selective destocking programmes by the national governments can also alleviate cash shortages for pastoralists (Jensen et al. 2017). Efforts to support pastoralists’ access to markets and food and income nutrition must adhere to COVID-19 PHMs as well as to the Humanitarian Charter and minimum standards for emergency responses (Sphere Association 2018; LEGS 2014; SEEP 2017). However, participatory assessments used for the design of interventions must be modified for and adhere to COVID-19 public health measures.

Gender-related differences in vulnerability are influenced by asset ownership and income, risk preferences, and cultural and social norms amongst men and women (Flintan 2008). It is therefore important to understand the different, but complementary, roles of men and women in pastoralist systems to identify how the pandemic will impact them. Table 6 summarizes gender roles in pastoral systems and anticipated gendered impacts of the pandemic. While globally men have a higher mortality rate from COVID-19 compared to women, possibly due to more prevalent co-morbidities (e.g. cardiovascular and lung disease), socio-cultural barriers in pastoral contexts result in women and girls having less access to healthcare than men and boys, resulting in health disparities that will likely be exasperated by COVID-19 (The Lancet 2020). With sick men and children in the household, women may adopt additional caregiver tasks, take on traditionally male-gendered livelihood tasks, and adopt social tasks as COVID-19 spreads through a community affecting multiple households. Health and livelihood support measures should consider the existing gender dynamics in pastoral communities and adapt accordingly (e.g. food assistance and supplementary animal fodder for women can help to meet household and livestock nutrition needs). Women and children are also at increased risk of domestic violence during pandemics (Peterman et al. 2020). Policy options to combat domestic violence include bolstering response systems and expanding social safety nets (Peterman et al. 2020) (Table 6).

**Table 6:** Existing gender dynamics amongst pastoralists with predicted impacts of COVID-19 (Flintan 2008; Kristjanson et al. 2014)

Gender category	Existing gender dynamics	Impacts of COVID-19
Men	• Traditional rights and ownership over livestock (variation due to local context) • Livestock management (e.g. purchasing, sale, disposal, or change) • Herding and grazing • Diagnosis and choice of treatment (ethno-veterinary knowledge)	• Increased risk of mortality • Men may need to take over food provision and livestock management activities generally carried out by women • More power for women to make decisions can threaten men who can feel marginalized
Women	• Give approval to livestock management activities • Managing and caring for animals near the household • Milking animals and deciding how much milk will be allocated for home consumption versus sale • Small ruminant ethno-veterinary knowledge	• Less access to healthcare with potentially worse COVID-19 health outcomes than men and boys • Women may need to take over herding and grazing responsibilities and act as the “head of the household” • Decreased ability to provide food due to limited market access and decreased ability to sell animal products • Increased work gathering wild foods to supplement nutrition • Increased work and risk of morbidity taking care of sick family and community members

## Conclusion

Responding to COVID-19 in GHA ASALs requires understanding the dynamics that are inherent to these pastoral systems, identifying successful health intervention strategies, and examining how ongoing shocks and vulnerabilities will interact with COVID-19 epidemiology and control measures. Currently, the direct health impacts of the virus on GHA ASALs appear limited, but little is known about its potential spread. Thus, our recommendations focus on disease prevention and understanding the epidemiology that will allow for a more effective allocation of resources. Based on our analysis of how COVID-19 control efforts will negatively impact pastoral livelihoods, food, income, and nutrition security, we recommend targeting high transmission risk areas and behaviours with public health measures to protect market access and ensure mobility. Food, income, and nutrition security and livelihood support measures for the GHA’s pastoralists that adhere to COVID-19 public health measures should be prioritized, along with public health interventions. Our paper is specific to the GHA. However, it can provide suggestions on how to contextualize the COVID-19 response to pastoralists in other parts of the world through a community-based approach that enables mobility and market access, while considering factors influencing pastoralist health and livelihoods in their totality (Bisson 2020).

## Data Availability

Not applicable

## Abbreviations

CAHWs: Community-based animal health workers
CBO: Community-based organization
CDC: Centres for Disease Control and Prevention
CDRs: Community disease reporters
CHWs: Community health workers
COHT: Community One Health team
COVID-19: Coronavirus disease 2019
FAO: Food and Agricultural Organization of the United Nations
FINS: Food, income, and nutrition security
GDP: Gross domestic product
GHA: Greater Horn of Africa
HTRA: High transmission risk area
HTRB: High transmission risk behaviour
MTN: Mobile Telecommunication Network
NGO: Non-governmental organization
PCD: Participatory community dialogue
PHMs: Public health measures
PRRAs: Preparedness, readiness, and response actions
RCCE: Risk communication and community engagement
SMS: Short message service
TTCL: Tanzania Telecommunication Corporation
WHO: World Health Organization
